# Supplementary material for: Fish Oil, but Not Olive Oil, Ameliorates Depressive-Like Behavior and Gut Microbiota Dysbiosis in Rats under Chronic Mild Stress
Source: Biomolecules. 2019 Sep 21;9(10):516. doi: 10.3390/biom9100516 (PMC6843453; doi:10.3390/biom9100516)
Supplement: Supplementary file 1 [file biomolecules-09-00516-s001.pdf]

Supplementary figure legends

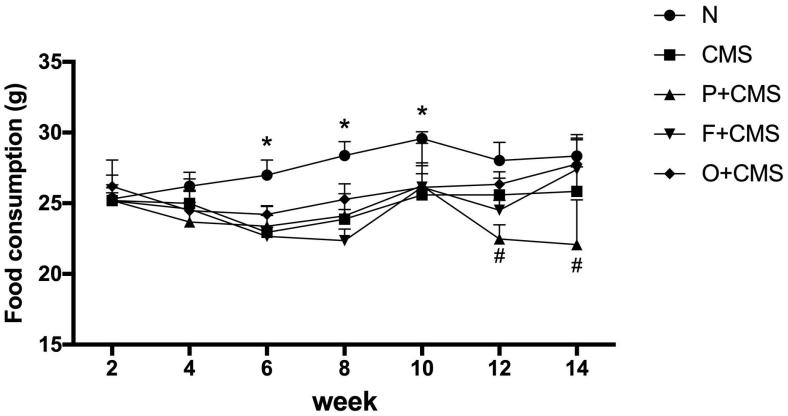

Figure. 1 Food consumption in the normal (N); chronic mild stress (CMS); CMS+imipramine (CMS+P); CMS+fish oil (CMS+F); and CMS+olive oil (CMS+O) groups. CMS was implemented from week 2 and significantly decreased the food consumption in weeks 4–6. Data are expressed as mean ± SD (n = 8 or 9 per group). * Significantly elevated in the N group compared with other groups, as determined using analysis of variance ($p < 0.05$); # Significantly decreased in the P+CMS group compared with other groups, as determined using analysis of variance ($p < 0.05$).

Supplementary table 1. Selected fatty acid profiles of different brain regions

Fatty acids profile (%)	Groups				
	**N**	**CMS**	**P+CMS**	**F+CMS**	**O+CMS**
**PFC**					
**SFA**					
C14:0	2.91 ± 3.19	2.33 ± 1.80	2.35 ± 1.68	2.75 ± 3.82	2.51 ± 2.59
C16:0	35.1 ± 5.7	37.2 ± 4.5	32.7 ± 8.0	28.6 ± 10.1	34.9 ± 7.8
C18:0	18.3 ± 8.9[b]	22.4 ± 6.9[ab]	25.6 ± 5.8[ab]	20.7 ± 5.0[ab]	29.5 ± 10.1[a]
**MUFA**					
C16:1	0.83 ± 1.02	0.89 ± 1.08	0.67 ± 0.33	2.55 ± 5.29	1.42 ± 2.55
C18:1 (OA)	12.7 ± 5.6	11.5 ± 2.2	11.8 ± 4.9	11.3 ± 3.2	9.15 ± 5.8
**PUFA**					
C18:2	1.43 ± 1.47	1.21 ± 1.18	1.22 ± 1.16	0.71 ± 0.46	0.89 ± 1.20
C20:4 (AA)	7.35 ± 2.14	8.43 ± 2.54	7.59 ± 4.39	7.87 ± 2.30	6.55 ± 4.17
C22:4	5.96 ± 2.30	5.27 ± 1.68	4.65 ± 3.46	5.04 ± 2.81	3.02 ± 2.00
C18:3 (ALA)	1.59 ± 1.71	0.88 ± 0.76	1.11 ± 0.62	1.62 ± 2.21	0.88 ± 0.56
C20:5 (EPA)	0.37 ± 0.59[b]	0.57 ± 0.65[b]	0.65 ± 0.26[b]	2.56 ± 2.29[a]	0.82 ± 1.11[ab]
C22:5 (DPA)	0.0 ± 0.0	0.0 ± 0.0	0.51 ± 0.59	0.76 ± 1.46	0.77 ± 1.95
C22:6 (DHA)	13.4 ± 8.7	9.13 ± 3.11	11.1 ± 5.7	13.5 ± 4.9	9.51 ± 7.20
n-6/n-3 ratio	1.19 ± 0.41	1.50 ± 0.33	0.97 ± 0.18	0.73 ± 0.21	0.90 ± 0.26
**Hippocampus**					
**SFA**					
C14:0	0.58 ± 0.47[b]	0.73 ± 0.20[b]	0.63 ± 0.08[b]	1.74 ± 1.31[ab]	2.26 ± 0.92[a]
C16:0	37.7 ± 2.9	36.7 ± 3.9	40.4 ± 5.3	34.5 ± 7.1	33.1 ± 1.2
C18:0	28.2 ± 1.1[a]	27.5 ± 2.0[ab]	29.7 ± 1.8[a]	27.7 ± 2.7[ab]	25.4 ± 1.2[b]
**MUFA**					
C16:1	0.24 ± 0.05	0.26 ± 0.07	0.32 ± 0.24	0.29 ± 0.38	0.34 ± 0.34
C18:1 (OA)	15.2 ± 3.0	14.3 ± 3.4	10.4 ± 6.0	12.2 ± 7.5	16.9 ± 1.3
**PUFA**					
C18:2	0.71 ± 0.43	0.86 ± 0.33	0.55 ± 0.35	0.51 ± 0.21	1.03 ± 1.19
C20:4 (AA)	5.30 ± 0.36	5.99 ± 2.11	5.51 ± 0.65	5.50 ± 1.42	6.72 ± 1.16
C22:4	4.38 ± 1.19	5.70 ± 1.31	4.30 ± 1.44	5.13 ± 2.15	3.88 ± 0.88
C18:3 (ALA)	0.44 ± 0.19	0.55 ± 0.25	0.58 ± 0.37	0.80 ± 0.43	0.93 ± 0.73
C20:5 (EPA)	1.23 ± 0.41[ab]	0.99 ± 0.54[b]	1.07 ± 0.60[b]	1.95 ± 0.68[a]	0.85 ± 0.36[b]
C22:5 (DPA)	0.05 ± 0.14	0.0 ± 0.0	0.0 ± 0.0	0.34 ± 0.69	0.77 ± 1.14
C22:6 (DHA)	6.01 ± 0.57[b]	6.42 ± 1.56[ab]	6.60 ± 1.34[ab]	9.32 ± 3.34[a]	7.88 ± 1.16[ab]
n-6/n-3 ratio	1.35 ± 0.16	1.69 ± 0.65	1.37 ± 0.31	0.97 ± 0.32	1.14 ± 0.74

N, normal group; CMS, chronic mild stress group; P+CMS, pharmaceutical + CMS group; F+CMS, fish oil + CMS group; O+CMS, olive oil + CMS group; SFA: saturated fatty acid; MUFA: monounsaturated fatty acid; PUFA: polyunsaturated fatty acid; OA: oleic acid; ALA: α-linolenic acid; AA: arachidonic acid; EPA: eicosapentaenoic acid; DPA: docosapentaenoic acid; DHA: docosahexaenoic aicd. Data are expressed as means (%) ± SD (n = 8-9/group). Values with different superscript letters mean significantly different at $p < 0.05$.

Supplementary table 2. Selected fatty acid profiles of RBC

Fatty acids profile (%)	Groups				
	**N**	**CMS**	**P+CMS**	**F+CMS**	**O+CMS**
**RBC**					
**SFA**					
C14:0	1.35 ± 0.84	0.94 ± 0.53	0.82 ± 0.72	1.21 ± 1.07	1.01 ± 0.71
C16:0	57.3 ± 8.3	56.5 ± 11.5	53.5 ± 9.3	62.2 ± 7.0	54.2 ± 10.0
C18:0	17.4 ± 3.5	17.2 ± 2.0	16.6 ± 1.9	14.4 ± 2.0	20.4 ± 7.5
**MUFA**					
C16:1	0.22 ± 0.25	0.13 ± 0.14	0.26 ± 0.21	0.54 ± 0.75	0.38 ± 0.51
C18:1 (OA)	7.72 ± 4.49	5.80 ± 3.27	6.42 ± 3.03	6.57 ± 2.76	7.32 ± 5.45
**PUFA**					
C18:2	3.79 ± 1.73	4.04 ± 2.18	5.23 ± 1.56	4.30 ± 1.42	3.58 ± 1.94
C20:4 (AA)	6.11 ± 4.18	8.11 ± 5.85	8.74 ± 4.38	3.55 ± 1.03	7.11 ± 5.13
C22:4	3.48 ± 2.52	4.12 ± 2.34	5.22 ± 2.50	3.51 ± 2.12	2.72 ± 1.85
C18:3 (ALA)	0.47 ± 0.51	0.32 ± 0.38	0.28 ± 0.19	0.48 ± 0.43	0.60 ± 0.63
C20:5 (EPA)	0.13 ± 0.14^{b}	0.25 ± 0.37^{b}	0.10 ± 0.11^{b}	0.67 ± 0.49^{a}	0.24 ± 0.23^{b}
C22:5 (DPA)	0.66 ± 0.84	1.20 ± 0.99	1.14 ± 0.66	1.15 ± 0.74	1.07 ± 1.30
C22:6 (DHA)	1.38 ± 0.96	1.28 ± 0.87	1.74 ± 1.16	1.35 ± 0.55	1.36 ± 1.19
n-6/n-3 ratio	5.36 ± 1.84	5.55 ± 1.76	6.72 ± 2.07	3.82 ± 1.99	6.97 ± 5.16

N, normal group; CMS, chronic mild stress group; P+CMS, pharmaceutical + CMS group; F+CMS, fish oil + CMS group; O+CMS, olive oil + CMS group; SFA: saturated fatty acid; MUFA: monounsaturated fatty acid; PUFA: polyunsaturated fatty acid; OA: oleic acid; ALA: α-linolenic acid; AA: arachidonic acid; EPA: eicosapentaenoic acid; DPA: docosapentaenoic acid; DHA: docosahexaenoic aicd. Data are expressed as means (%) ± SD (n = 8-9/group). Values with different superscript letters mean significantly different at $p < 0.05$.
